# Atypical teratoid/rhabdoid tumors (ATRTs) with *SMARCA4* mutation are molecularly distinct from *SMARCB1*-deficient cases

**DOI:** 10.1007/s00401-020-02250-7

**Published:** 2020-12-17

**Authors:** Dörthe Holdhof, Pascal D. Johann, Michael Spohn, Michael Bockmayr, Sepehr Safaei, Piyush Joshi, Julien Masliah-Planchon, Ben Ho, Mamy Andrianteranagna, Franck Bourdeaut, Annie Huang, Marcel Kool, Santhosh A. Upadhyaya, Anne E. Bendel, Daniela Indenbirken, William D. Foulkes, Jonathan W. Bush, David Creytens, Uwe Kordes, Michael C. Frühwald, Martin Hasselblatt, Ulrich Schüller

**Affiliations:** 1 Department of Pediatric Hematology and Oncology, University Medical Center Hamburg-Eppendorf, Hamburg, Germany; 2 Research Institute Children’s Cancer Center Hamburg, Martinistrasse 52, N63 (HPI), 20251 Hamburg, Germany; 3 Paediatric and Adolescent Medicine, Swabian Childrens’ Cancer Center Augsburg, Augsburg, Germany; 4 Hopp Children’s Cancer Center (KiTZ), Heidelberg, Germany; 5 Division of Pediatric Neurooncology, German Cancer Research Center (DKFZ) and German Cancer Research Consortium (DKTK), Heidelberg, Germany; 6 Institute of Pathology, Corporate Member of Freie Universität Berlin, Charité, Universitätsmedizin Berlin, Humboldt-Universität Zu Berlin and Berlin Institute of Health, Berlin, Germany; 7 Institute of Neuropathology, University Medical Center Hamburg-Eppendorf, Hamburg, Germany; 8 INSERM U830, Laboratory of Translational Research in Pediatric Oncology, SIREDO Pediatric Oncology Center, Curie Institute, Paris, France; 9 Division of Hematology and Oncology, Arthur and Sonia Labatt Brain Tumour Research Centre, The Hospital for Sick Children, Department of Paediatrics, University of Toronto, Toronto, ON Canada; 10 INSERM U900, CBIO-Centre for Computational Biology, MINES ParisTech, PSL Research University, Curie Institute, Paris, France; 11 Departments of Genetics and of Oncopediatry and Young Adults, Curie Institute, Paris, France; 12 Princess Máxima Center for Pediatric Oncology, Utrecht, The Netherlands; 13 Department of Oncology, St Jude Children’s Research Hospital, Department of Pediatrics, University of Tennessee Health Sciences Center, Memphis, TN USA; 14 Children’s Hospitals and Clinics of Minnesota, Minneapolis, MN USA; 15 Heinrich-Pette Institute, Leibniz Institute for Experimental Virology, Hamburg, Germany; 16 Department of Human Genetics, McGill University, Montreal, QC Canada; 17 Division of Anatomical Pathology, British Columbia Children’s Hospital and Women’s Hospital and Health Center, Vancouver, BC Canada; 18 University of British Columbia, Vancouver, BC Canada; 19 Department of Pathology, Ghent University Hospital, Ghent, Belgium; 20 Institute of Neuropathology, University Hospital Münster, Münster, Germany

**Keywords:** SMARCA4, BRG1, ATRT, Rhabdoid, DNA methylation, RNA sequencing

## Abstract

Atypical teratoid/rhabdoid tumors (ATRTs) are very aggressive childhood malignancies of the central nervous system. The underlying genetic cause are inactivating bi-allelic mutations in *SMARCB1* or (rarely) in *SMARCA4.* ATRT-SMARCA4 have been associated with a higher frequency of germline mutations, younger age, and an inferior prognosis in comparison to *SMARCB1* mutated cases. Based on their DNA methylation profiles and transcriptomics, *SMARCB1* mutated ATRTs have been divided into three distinct molecular subgroups: ATRT-TYR, ATRT-SHH, and ATRT-MYC. These subgroups differ in terms of age at diagnosis, tumor location, type of *SMARCB1* alterations, and overall survival. ATRT-SMARCA4 are, however, less well understood, and it remains unknown, whether they belong to one of the described ATRT subgroups. Here, we examined 14 ATRT-SMARCA4 by global DNA methylation analyses. We show that they form a separate group segregating from *SMARCB1* mutated ATRTs and from other *SMARCA4*-deficient tumors like small cell carcinoma of the ovary, hypercalcemic type (SCCOHT) or *SMARCA4* mutated extra-cranial malignant rhabdoid tumors. In contrast, medulloblastoma (MB) samples with heterozygous *SMARCA4* mutations do not group separately, but with established MB subgroups. RNA sequencing of ATRT-SMARCA4 confirmed the clustering results based on DNA methylation profiling and displayed an absence of typical signature genes upregulated in *SMARCB1* deleted ATRT. In summary, our results suggest that, in line with previous clinical observations, ATRT-SMARCA4 should be regarded as a distinct molecular subgroup.

**Supplementary Information:**

The online version contains supplementary material available at 10.1007/s00401-020-02250-7.

## Introduction

Malignant rhabdoid tumors (MRTs) are highly aggressive malignancies usually affecting young children and infants. They may occur in any part of the body, but the majority (66%) is detected in the central nervous system (CNS), where they are called atypical teratoid/rhabdoid tumors (ATRT) [9]. With an incidence of 1.4 per million in Germany [48], ATRT is a rare tumor entity even in pediatric oncology. Still, it is the most common embryonal CNS tumor in children younger than 12 months [36, 48]. Independent of tumor location, loss of function mutations in components of the SWItch/Sucrose Non-Fermentable (SWI/SNF) chromatin remodeling complex are a characteristic feature and represent the sole recurrent genetic alteration in all MRTs [16, 17, 29]. In the vast majority of MRTs, pathogenic variants (hereafter “mutations”) affect the *SMARCB1* gene. In rare cases (about 0.5–2% of ATRT [12, 22]), *SMARCA4* is mutated instead [17, 42, 43]. Since these mutations result in loss of the respective protein, loss of staining for either SMARCB1 or SMARCA4 by immunohistochemistry is used as a diagnostic tool to ensure the diagnosis of an ATRT [30]. About one-third of patients with *SMARCB1*-deficient MRTs carry germline mutations within the *SMARCB1* gene [3, 7, 20]. Although based on very small numbers, it appears as if patients with an ATRT-SMARCA4 are even more often carriers of germline mutations. Furthermore, the latter group of patients is younger and, as their survival is even shorter, their tumors seem to be even more aggressive [17, 20]. However, due to the small number of patients suffering from an ATRT-SMARCA4, accompanied by the lack of suitable in vitro and in vivo models, knowledge regarding biological mechanisms involved in tumor development is limited. Besides MRTs, a role of SMARCA4 has been described for a variety of tumor entities including non-small cell lung cancer or thoracic sarcomas [28, 39]. In small cell carcinoma of the ovary, hypercalcemic type (SCCOHT), loss of the SMARCA4 protein has been proposed to be the main driving event in tumorigenesis [46]. In other tumor types, such as Burkitt lymphoma or medulloblastoma (MB), heterozygous missense mutations in *SMARCA4* have been identified, but their role in tumor development remains elusive [23, 26, 38, 41].

Recently, DNA methylation profiling has become an attractive asset in the diagnostics of CNS tumors [4]. Based on global DNA methylation and/or gene expression, ATRTs can be divided into three distinct subgroups [15, 22, 47], designated as ATRT-TYR, ATRT-SHH, and ATRT-MYC [19]. The subgroups differ in the expression of distinct genes, the activation of specific signaling pathways, and in clinical parameters. For instance, ATRT-TYR, especially when older than 12 months, have been associated with a slightly better prognosis compared to the other subgroups or younger patients [12]. In addition to the subgrouping of ATRTs, extra-cranial MRTs (eMRTs) can also be further divided into subgroups based on molecular characteristics [5, 6]. Of note, DNA methylation profiles of eMRTs share many characteristics with ATRT-MYC.

Although these studies helped to increase the knowledge of the disease, they were predominantly based on *SMARCB1* mutated ATRTs and eMRTs. Hitherto, three *SMARCA4*-deficient cases were included in one of the studies [22] and clustered to ATRT-SHH. However, it remains unknown, if ATRT-SMARCA4 belong to this subgroup in general, if they are divided into the same subgroups as *SMARCB1*-deficient ones, or, if they make up their own subgroup. To address these questions, we examined DNA methylation profiles and RNA sequencing data. Our results suggest that ATRT-SMARCA4 should be regarded as a separate molecular ATRT subgroup distinct from ATRT-MYC, ATRT-SHH, and ATRT-TYR.

## Materials and methods

### Tumor samples

We used published and unpublished data sets for all analyses presented here (Supplementary Table 1, online resource). Clinical data for previously unreported cases are summarized in Table 1 as well as in Supplementary Tables 2 and 3, online resource. DNA methylation or gene expression data from published data sets are available at the gene expression omnibus (GEO), accession numbers GSE70678, GSE90496, and GSE123601 and/or in the original publications [4–6, 8, 22, 34, 47]. Diagnosis of the respective tumor entity was assured by a (neuro) pathologist. To characterize the individual *SMARCA4* mutations, i.e., predict the respective amino acid change, coding impact, and clinical significance/pathogenicity, we used the search engine VarSome [25]. Predicted pathogenicity was based on ClinVar [27] and The American College of Medical Genetics and Genomics (ACMG) classification [40]. Survival of *SMARCB1* and *SMARCA4* mutated ATRTs was compared by performing a log-rank (Mantel-Cox) test using the Prism Software Version 7 (GraphPad Software, Inc. San Diego, USA).

**Table 1:** Overview of ATRT-SMARCA4 included in this study

Case No	Tumor location	Sex	Age at diagnosis [months]	Overall survival [months]	Dead/ alive	Brain Tumor Classifier Result (score)	*SMARCA4* mutation in the tumor	Predicted AA change	Coding impact	Clinical significance (ClinVar)	Clinical significance (ACMG)	*SMARCA4* germline mutation	DNA methylation data	RNA seq data	SMARC4 protein (IHC)	References
01	supra	Female	3	1	Dead	No match^a^	c.3407delG	p.G1136Afs*4	Frameshift	n/a	Pathogenic	Yes	√	√	Lost	Patient #8 [17]
02	supra	Male	11	n/a	N/a	No match^a^	c.1666C > T^b^	p.Q556*	Nonsense	Pathogenic	Pathogenic	n/a	√	√	Lost	
03	supra	Female	11	2	Dead	No match^a^	c.352C > T	p.Q118*	Nonsense	n/a	Pathogenic	Yes	√	√	Lost	
04	supra	Male	3	n/a	Dead	ATRT-SHH (0.99)	Breakage within the *SMARCA4* region (FISH)	-	n/a	n/a	n/a	n/a	√	x	Lost	
05	infra	Female	10	2	Dead	ATRT-SHH (0.99)	c.3565C > T	p.R1189*	Nonsense	Pathogenic	Pathogenic	Yes^c^	√	x	Lost	Patient #1 [17] Patient III2 [43]
06	infra	Female	7	1	Dead	ATRT-SHH (0.98)	c.4038_4075del38	p.I1347*	Nonsense	n/a	Pathogenic	Yes	√	x	Lost	Patient #4 [17]
07	supra, Multiple	Male	22	3	Dead	No match^a^	Heterozygous *SMARCA4* deletion (FISH)	-	n/a	n/a	n/a	no	√	√	Lost	Patient #5 [17]
08	infra	Male	17	28	Dead	No match^a^	c.2335G > A	p.D779N	Missense	Uncertain significance	Uncertain significance	Yes	√	x	Retained	Patient #9 [17]
09	supra, metastatic	Male	3	1	Dead	ATRT-SHH (0.94)	c.2920delC	p.F975Sfs*44	Frameshift	Pathogenic	Pathogenic	Yes	√	x	Lost	
10	infra	Female	46	21	Dead	ATRT-SHH (0.92)	c.3574C > T	p.R1192C	Missense	Uncertain significance	Uncertain significance	no	√	√	Retained	[32]
11	supra	Male	1	n/a	Dead	No match^a^	n/a	n/a	n/a	n/a	n/a	n/a	√	x	Lost	
12	infra	Male	13	22	Alive	No match^a^	c.1757_1760del	p.K586Rfs*26	Framehsift	Pathogenic	Pathogenic	n/a	√	√	Lost	
13	supra	Male	4	9	Alive	ATRT-SHH (0.96)	c.3168 + 5G > A	*Intron*	None	n/a	Uncertain significance	n/a	√	√	Lost	
14	supra	Male	3	3	Dead	ATRT-SHH (0.99)	c.3277C > T	p.R1093*	Nonsense	Likely Pathogenic	Pathogenic	n/a	√	√	Lost	

^a^Interpreted by the brain tumor classifier (www.molecularneuropathology.org)

^b^Inferred from RNA Seq data

^c^Inferred since the same *SMARCA4* mutation found in the patient’s tumor was found in her sister’s and father’s germline; infra infratentorial, n/a data not available, supra supratentorial, √ analysis done, x analysis not done

### DNA methylation profiling

For DNA isolation from FFPE tissue, 10 × 10 µm sections were cut and DNA isolated using the ReliaPrep™ FFPE gDNA Miniprep System (Promega) according to manufacturer’s instructions. About 100–500 ng DNA was used for bisulfite conversion by the EZ DNA Methylation Kit (Zymo Research). Afterwards, the DNA Clean & Concentrator-5 (Zymo Research) and the Infinium HD FFPE DNA Restore Kit (Illumina) were employed to clean and restore the converted DNA. Finally, either the HumanMethylation450 BeadChip array or the Infinium MethylationEPIC BeadChip Kit (both Illumina) were used to quantify the methylation status of 450,000 or 850,000 CpG sites, respectively, on an iScan device (Illumina).

### RNA sequencing

For RNA isolation from FFPE tissue, 10 × 10 µm sections were cut and RNA isolated using the Maxwell^®^ RSC RNA FFPE Kit (Promega). The RNA integrity was analyzed with the RNA 6000 Nano Chip on an Agilent 2100 Bioanalyzer (Agilent Technologies). From total RNA, the ribosomal RNA was depleted with the help of the RiboCop rRNA Depletion Kit (Lexogen) followed by RNA sequencing library generation using the CORALL Total RNA-Seq Library Prep Kit (Lexogen). Concentrations of all samples were measured with a Qubit 2.0 Fluorometer (Thermo Fisher Scientific) and fragment lengths distribution of the final libraries was analyzed with the DNA High Sensitivity Chip on an Agilent 2100 Bioanalyzer (Agilent Technologies). All samples were normalized to 2 nM and pooled at equimolar concentrations. The library pool was sequenced on the NextSeq500 (Illumina) with 1 × 75 bp, with 24.5–35.1 million reads per sample.

### Bioinformatics

**Fig. 1:**
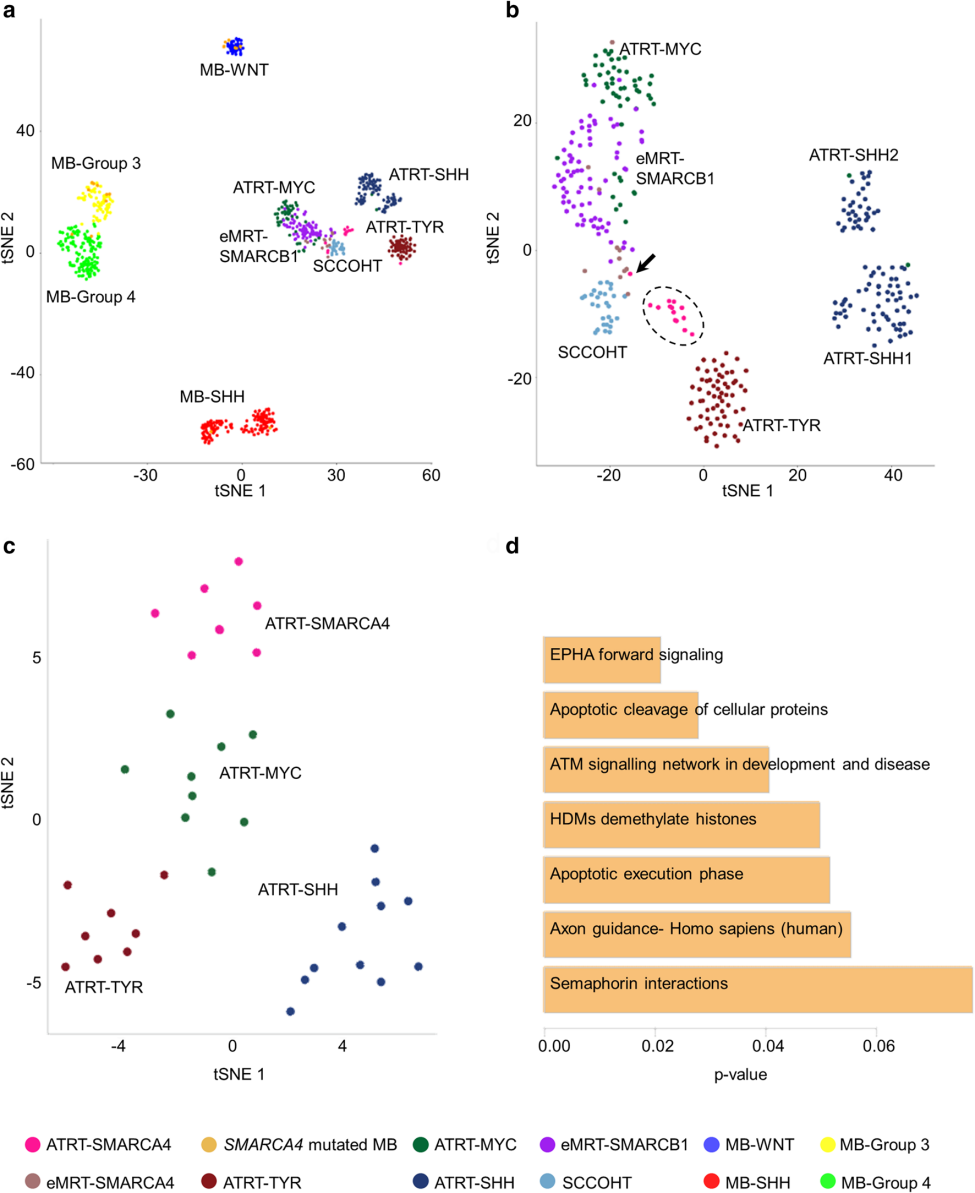
Unsupervised *t*-SNE analysis based on the 40,426 most variant probes of DNA methylation profiles of ATRT-SMARCA4 (*n* = 14, pink), eMRT-SMARCA4 (*n* = 11, brown), MBs with *SMARCA4* mutation (*n* = 18, ocher), *SMARCB1* mutated ATRTs [*n* = 62 ATRT-TYR (dark red), *n* = 93 ATRT-SHH (blue), *n* = 53 ATRT-MYC (dark green)], eMRT-SMARCB1 (*n* = 74, purple), *SMARCA4* wild-type MBs [*n* = 37 MB-WNT (dark blue), *n* = 135 MB-SHH (red), *n* = 75 MB-Group 3 (yellow), *n* = 138 MB-Group 4 (green)] and SCCOHTs (*n* = 28, light blue) (**a**). Unsupervised *t*-SNE analysis based on the 14,772 most variant probes of DNA methylation profiles of the same samples but without MB samples (**b**). Unsupervised *t*-SNE analysis based on the 1000 most variable genes across all tumor samples, i.e., *n* = 8 ATRT-SMARCA4 (pink), *n* = 8 ATRT-TYR (red), *n* = 11 ATRT-SHH (blue) and *n* = 9 ATRT-MYC (green), Of note, ATRT-SMARCA4 cases 10 and 13 displayed strong similarities, as their data points almost overlap on the *t*-SNE plot (**c**). Significantly enriched pathways in ATRT-SMARCA4 are sorted according to *p*-values and depicted in a bar chart (**d**). Arrow indicates case 7

IDAT files were processed as previously described [45]. In detail, raw data files were loaded into R using the minfi package (v.1.32.0). Since we included data derived from EPIC and 450 K arrays, we used single-sample normalization method (ssNoob) [10] for normalization of all samples. Furthermore, we excluded probes targeting the sex chromosomes. For *t*-distributed stochastic neighbor embedding (*t*-SNE) analysis, the Rtsne (v.0.15) package was employed. Probes were selected by standard deviation > 0.25, resulting in 40,426 probes for Fig. 1a and 14,772 probes for Fig. 1b. Perplexity was set to 28 and 19 for Fig. 1a, b, respectively. The heatmap were build based on the 1000 most variable probes by standard deviation with R package pheatmap (v1.0.12, https://CRAN.R-project.org/package=pheatmap), using the clustering method “ward.D2”. Global DNA methylation levels were calculated as described in [22] and significant differences determined by a Wilcoxon Rank Sum Test. Genome-wide chromosomal losses and gains as well as gains and losses at the *SMARCA4* locus were calculated as published previously [6, 37].

Fastq files from single end or paired end sequencing were aligned to hg19 human genome using STAR aligner (v2.5.2a). Read counts were obtained from the STAR generated BAM files with featureCounts (v2.0.0) using hg19 gene annotation (Ensembl release 87). The count matrix was used to obtain normalized reads via variance stabilizing transformation applied through DESeq2 R package (v1.26.0) and then batch corrected using removeBatchEffect function of R package limma (v3.42.2). Batch corrections was performed between sequencing data generated from FFPE samples (the *SMARCA4*-deficient samples) and the data derived from FF samples (*SMARCB1* samples).

For *t*-SNE analysis, we used the packages R-tsne and employed only the 500, 1000, and 2000 most variable genes throughout all samples. Perplexity was set to 30 and theta to 0.5.

For differential expression analysis, we used the DESeq2 R package and performed correction for multiple testing using the Benjamini Hochberg method. For boxplots of meaningful, differentially expressed genes, we performed ANOVA analysis with post-hoc contrast testing using the built in aov function of R. For the genes *MITF*, *MYC,* and *GLI2*—upregulated only in one of the ATRT subgroups—we used a t.test of ATRT-SMARC4 versus the subgroups ATRT-TYR, ATRT-MYC, and ATRT-SHH. For pathway enrichment analysis, we employed the ConensusPathDB tool (http://cpdb.molgen.mpg.de/) using the “enrichment analysis” option and default datasets to be tested. Visualization was performed using the ClueGo tool (version 2.5.7) from Cytoscape using default parameters.

### Data availability

The DNA methylation and gene expression data of this study have been deposited in NCBI’s Gene Expression Omnibus (GEO; http://www.ncbi.nlm.nih.gov/geo) and are accessible through GEO Series accession numbers GEO: GSE161692 and GSE160748, respectively.

## Results

### ATRT-SMARCA4 do not group with the known ATRT subtypes based on DNA methylation pattern

In this study, we included 8 unpublished and six previously reported ATRT samples with a verified *SMARCA4* alteration [17, 32, 43]. All clinical data including genetics and survival are depicted in Table 1 and Supplementary Fig. 1, online resource. We performed global DNA methylation analysis of all 14 tumor samples and used the Heidelberg Brain Tumor classifier (v11b4) [4] to validate, whether they matched to one of the established ATRT subgroups. Fifty percent of the samples (7/14) were classified (score > 0.9) as an ATRT-SHH, 21% (3/14) showed the best, but not a specific match with ATRTs (score = 0.58–0.87), whereas 29% (4/14) did not match to any methylation class at all. Aiming to better characterize our tumor samples in an unsupervised way, we performed *t*-SNE. To this end, we included previously published and unpublished DNA methylation profiles of other SWI/SNF-deficient tumors, i.e., SCCOHTs and eMRTs (Supplementary Table 1–2, online resource [5, 6, 8]). The latter comprised samples with either a *SMARCA4* or a *SMARCB1* mutation. Furthermore, we included MBs with heterozygous *SMARCA4* missense mutations as well as *SMARCA4* wild-type MBs and further ATRTs (Supplementary Table 3, online resource [4, 34]).

We recognized that MBs with *SMARCA4* mutations grouped with their annotated MB subgroups (Fig. 1a). ATRT-SMARCA4 located close to the SCCOHTs, ATRTs, and eMRTs without grouping distinctly to one tumor entity. We then removed all MB samples and recalculated the *t*-SNE (Fig. 1b) and additionally performed unsupervised hierarchical clustering (Supplementary Fig. 2, online resource). Thereby, we recognized that ATRT-SMARCA4 arranged as a separate class, whereas the eMRTs with *SMARCA4* deficiency were located in between the SCCOHTs and the *SMARCA4* wild-type eMRTs. Case 7 (arrow in Fig. 1b) grouped with *SMARCA4*-deficient eMRTs instead of ATRTs. However, this patient did not only harbor multiple supratentorial lesions, but also tumor masses at the left cardiac ventricle and in the right proximal femur. Therefore, we cannot rule out that case 7 is in fact the metastasis of an eMRT. Finally, ATRT-SMARCA4 had the lowest methylation levels of all ATRTs (Supplementary Fig. 3). Taken together, DNA methylation patterns of ATRT-SMARCA4 indicate that these tumors cannot be included in the established classification of *SMARCB1* mutated ATRTs.

### The transcriptome of ATRT-SMARCA4 is distinct from SMARCB1 altered ATRTs and suggests enhanced Ephrin signaling as a possible tumor driver

Next, we performed RNA sequencing of 8 ATRT-SMARCA4 (Table 1) and compared them to *SMARCB1* mutated ATRT by *t*-SNE analysis (Fig. 1c). We recognized four distinct subgroups in the *t*-SNE plot corresponding to ATRT-TYR, ATRT-SHH, ATRT-MYC, and ATRT-SMARCA4. As expected, *SMARCA4* expression levels were reduced, whereas *SMARCB1* expression levels were higher in SMARCA4-deficient tumor samples compared to all other ATRT subgroups (Supplementary Fig. 4a,b, online resource). *SMARCA2*, which is supposed to be post-transcriptionally downregulated in SCCOHTs [24], displayed lower expression values as well (Supplementary Fig. 4c, online resource). Besides these SWI/SNF-related genes, the *SMARCA4*-deficient samples featured high expression values of *EPHA5*, *ROCK1*, and *FGF10* as well as low expression levels of *GLI2, MITF, MYC,* and *DMRT2* (Supplementary Fig. 4d-j, online resource). Concordantly, functional analysis using ConsensusPathDB [18] identified *EPHA forward signaling* including the genes *EPHA5* and *ROCK1* as the most significant enriched pathway (Fig. 1d). As *EPHA5* is known to be significantly upregulated in SCCOHT as well [1], Ephrin signaling might be one of the tumor-promoting pathways in tumors driven by *SMARCA4* deficiency. Finally, network analysis suggested further processes including neuronal function associated terms such as *Neuroactive ligand-receptor interaction* and *Membrane depolarization* to be altered in ATRT-SMARCA4 (Supplementary Fig. 5, online resource). Overall, comparing the transcriptomes of *SMARCA4* and *SMARCB1* mutated ATRTs sustains the notion that the former forms a separate subgroup marked by Ephrin signaling rather than fitting into the established ATRT subclasses.

### SMARCA4 deficiency is not the result of large chromosomal deletions

The ATRT subgroups ATRT-TYR, ATRT-SHH, and ATRT-MYC are not only different in regard to their DNA methylation profiles and their transcriptomes, but also harbor different kinds of tumor-driving *SMARCB1* alterations [19]. To examine the genetic basis of *SMARCA4* deficiency in our cohort, we investigated chromosomal gains and losses (Supplementary Fig. 6, online resource) and alterations specifically at the *SMARCA4* locus located on chr19p13.2 (Supplementary Fig. 7, online resource). As expected, the only chromosomal alterations in *SMARCB1*-deficient ATRTs were losses on chromosome 22q, harboring *SMARCB1*. Deletions of chromosomal regions of 4q and 6q were detected in *SMARCB1* mutated eMRTs. Neither ATRTs nor eMRTs nor SCCOHTs displayed large chromosomal alterations, including chromosomal arm 19p. The *SMARCA4* locus revealed no deletions in any of the examined tumor samples.

## Discussion

In this study, we collected 14 samples of ATRT-SMARCA4 aiming at the molecular characterization of these tumors. Based on clinical data, our cohort fits well with all previously published cases [17], as the patients were very young infants, who died shortly after diagnosis. Since we were particularly interested in whether these tumors belong to one or more of the three known ATRT subgroups (ATRT-TYR, ATRT-SHH, ATRT-MYC), we performed DNA methylation and RNA sequencing analysis to examine them on a molecular level.

First, only half of our cases were recognized as ATRTs by the Heidelberg Brain Tumor classifier. The ones that were recognized matched all to ATRT-SHH. This was in line with the previously published observation that 3 *SMARCA4* mutant cases matched to ATRT-SHH in an unsupervised hierarchical clustering analysis of DNA methylation data [22]. Still, 50% of our cases could not be diagnosed using the Heidelberger classifier. This uncovers a problem in the diagnostics of these tumors, as the classifier has become an import neuropathological tool, in particular for rare and aggressive brain tumors. In *t*-SNE analyses, ATRT-SMARCA4 grouped overall together with *SMARCB1*-deficient RTs and SCCOHTs, but did not locate specifically within one of these tumor subclasses. Furthermore, they clustered apart from the established ATRT subgroups and formed a separate subcluster within the group of *SMARCA4*-deficient tumors. In contrast, MBs carrying heterozygous *SMARCA4* missense mutations, clearly grouped together with one of the four MB subgroups on *t*-SNE. This finding suggests that in ATRTs the *SMARCA4* alterations, which are either homozygous nonsense or homozygous missense mutations, have a major impact on the tumor’s methylome, whereas the heterozygous missense mutations in MB might be less important in this regard. SCCOHTs and eMRTs, especially those with *SMARCA4* deficiency, showed high similarities in our analyses, even though not being randomly intermingled. Our results are, therefore, in line with previously published studies discussing whether SCCOHTs shall be regarded as a subgroup of RTs [8, 11].

ATRTs and eMRTs with *SMARCA4* deficiency as well as *SMARCB1* mutated eMRTs and ATRT-MYC were hypomethylated and differed significantly from the hypermethylated ATRT-SHH and ATRT-TYR. This finding is in agreement with other studies that have previously investigated global methylation levels of *SMARCB1*-deficient RTs [6, 19]. Furthermore, SCCOHTs also displayed the lowest methylation levels, indicating that *SMARCA4*-deficient tumors demonstrate a rather hypomethylated phenotype. This may promote tumor development and progression by changing the global heterochromatin structure and activating proto-oncogenes or germline specific genes [31, 49]. Furthermore, global hypomethylation is associated with a poor prognosis in cancer entities, such as myeloma or type I ovarian cancers [35, 44] and increases during the malignant transformation of meningioma [13]. This might be associated with the even more aggressive behavior of ATRT-SMARCA4 compared to *SMARCB1* mutated ones.

We identified possible signature genes for ATRT-SMARCA4, as *EPHA5*, *ROCK1,* and *FGF10* were up- and *DMRT2* significantly downregulated in these tumors. The most significant enriched pathway was *Ephrin forward signaling* that is important for embryonic CNS development and also known to be altered in different cancer entities such as glioma [33]. As several pharmacological tools for the inhibition of Ephrin signaling including those targeting EPHA5 have been tested for other diseases, this might be a good starting point for future studies on ATRT-SMARCA4 [14, 21, 50].

**Fig. 2:**
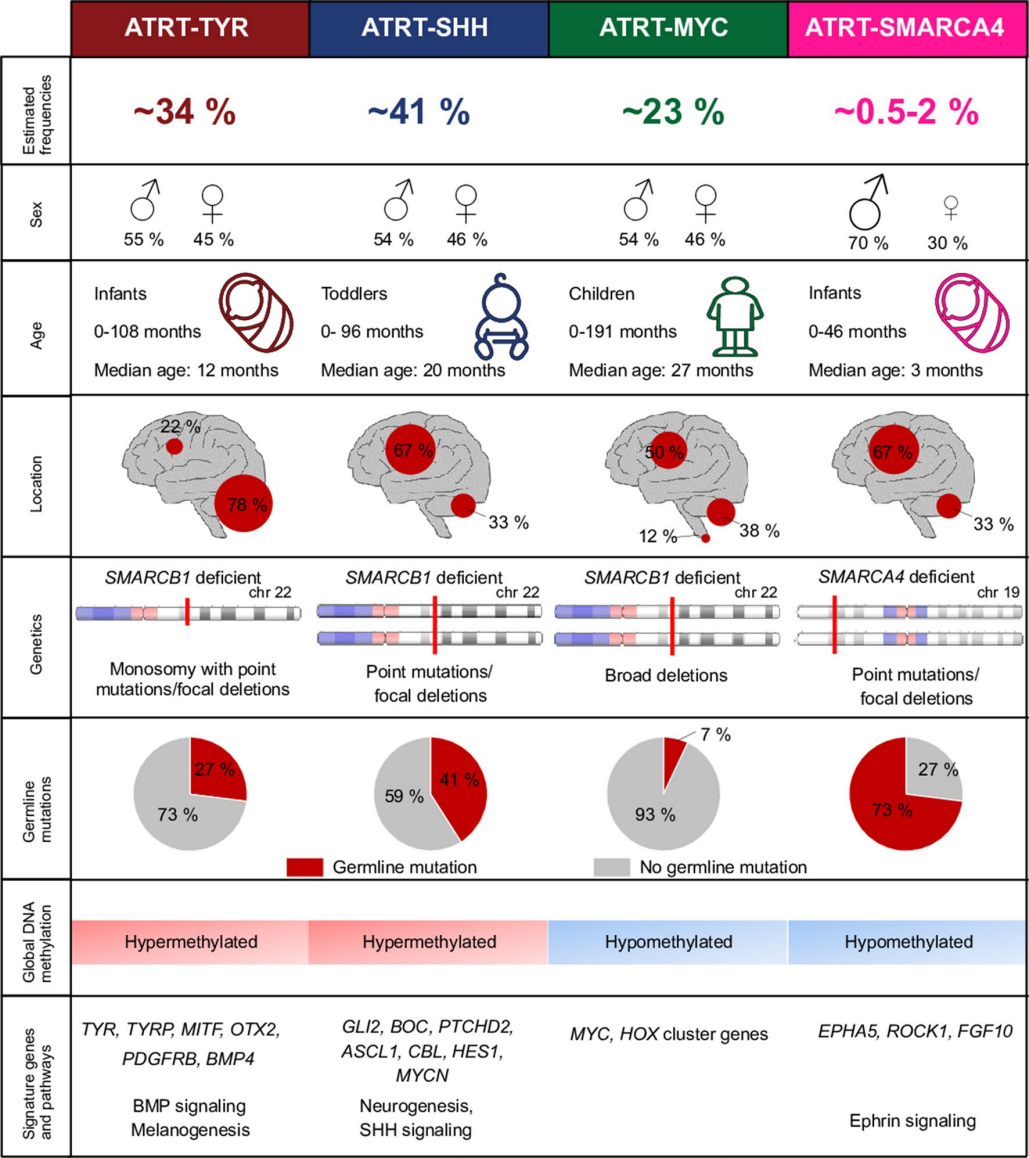
Proposed model for including ATRT-SMARCA4 in the subgroup classification of ATRTs. Note that frequencies for each subgroup are based on published datasets and represent only a rough estimation. Estimated frequencies of *SMARCB1*-deficient ATRT subgroups (*n* = 321), their male to female ratios (*n* = 82 for ATRT-TYR, *n* = 105 for ATRT-SHH, *n* = 56 for ATRT-MYC), age (*n* = 62 for ATRT-TYR, *n* = 72 for ATRT-SHH, *n* = 43 for ATRT-MYC), and locations (*n* = 68 for ATRT-TYR, *n* = 91 for ATRT-SHH, *n* = 48 for ATRT-MYC) as well as information regarding genetics, signature genes and pathways is based on the study by Ho et al. [19]. Frequencies of germline mutations was taken from Frühwald et al. [12]. Frequencies of ATRT-SMARCA4 is estimated based on studies published by Johann et al. [22] and Frühwald et al. [12]. Information concerning the sex ratio (*n* = 19), age (*n* = 19), location (*n* = 19), and germline mutations (*n* = 10) of ATRT-SMARCA4 are taken from the study presented here and published reports [2, 17]. Genetics, global DNA methylation levels as well as signature genes and pathways of ATRT-SMARCA4 are based on the here generated results. Design is inspired by the model proposed by Ho et al. [19]

Overall, based on the data presented here, we propose that ATRT-SMARCA4 should be regarded as a separate ATRT subgroup. As reported previously [17], they affect young children, who are often carriers of germline mutations. Additionally, the male predominance is even more pronounced and their global DNA methylation patterns as well as their global transcriptomics differ from the *SMARCB1* mutated ATRT subgroups. The data are summarized in Fig. 2, which gives an overview on ATRT subgroups including the here described new findings. In future studies, these findings might be used to adjust therapeutic options in the clinic and consequently improve the survival of patients.

## Supplementary Information

Below is the link to the electronic supplementary material.

Supplementary file1 (PDF 842 KB)
