# Supplementary material for: Atypical teratoid/rhabdoid tumors (ATRTs) with SMARCA4 mutation are molecularly distinct from SMARCB1-deficient cases
Source: Acta Neuropathol. 2020 Dec 17;141(2):291–301. doi: 10.1007/s00401-020-02250-7 (PMC7847432; doi:10.1007/s00401-020-02250-7)
Supplement: Supplementary file 1 — Supplementary file1 (PDF 842 KB) [file 401_2020_2250_MOESM1_ESM.pdf]

Title Page

Title

Atypical teratoid/rhabdoid tumors (ATRTs) with *SMARCA4* mutation are molecularly distinct from *SMARCB1* deficient cases

Authors

Dörthe Holdhof[1,2,#], Pascal D. Johann[3,4,5,#], Michael Spohn[2], Michael Bockmayr[1,2,6], Sepehr Safaei[1,2,7], Piyush Joshi[4,5], Julien Masliah-Planchon[8], Ben Ho[9], Mamy Andrianteranagna[8,10], Franck Bourdeaut[8,11], Annie Huang[8], Marcel Kool[4,5,12], Santhosh A. Upadhyaya[13], Anne E Bendel[14], Daniela Indenbirken[15], William D. Foulkes[16], Jonathan W. Bush[17,18], David Creytens[19], Uwe Kordes[1], Michael C. Frühwald[3], Martin Hasselblatt[20], and Ulrich Schüller[1,2,7]

[1]Department of Pediatric Hematology and Oncology, University Medical Center Hamburg-Eppendorf, Germany

[2]Research Institute Children's Cancer Center Hamburg, Germany

[3]Paediatric and Adolescent Medicine, Swabian Childrens' Cancer Center Augsburg, Germany

[4]Hopp Children's Cancer Center (KiTZ), Heidelberg, Germany.

[5]Division of Pediatric Neurooncology, German Cancer Research Center (DKFZ) and German Cancer Research Consortium (DKTK), Heidelberg, Germany.

[6]Institute of Pathology, Charité - Universitätsmedizin Berlin, corporate member of Freie Universität Berlin, Humboldt-Universität zu Berlin and Berlin Institute of Health, Berlin, Germany.

[7]Institute of Neuropathology, University Medical Center Hamburg-Eppendorf, Germany

[8]INSERM U830, Laboratory of Translational Research in Pediatric Oncology, SIREDO Pediatric Oncology Center, Curie Institute, Paris, France.

[9]Division of Hematology & Oncology, Arthur and Sonia Labatt Brain Tumour Research Centre, The Hospital for Sick Children, Department of Paediatrics, University of Toronto, Toronto, ON, Canada

[10]INSERM U900, MINES ParisTech, PSL Research University, CBIO-Centre for Computational Biology, Curie Institute, Paris, France

[11]Departments of Genetics and of Oncopediatry and Young Adults, Curie Institute, Paris, France.

[12]Princess Máxima Center for Pediatric Oncology, Utrecht, the Netherlands

[13]Department of Oncology, St Jude Children's Research Hospital, Department of Pediatrics, University of Tennessee Health Sciences Center, Memphis, Tennessee, USA

[14]Children's Hospitals and Clinics of Minnesota, Minneapolis, MN, USA

[15]Heinrich-Pette Institute, Leibniz Institute for Experimental Virology, Hamburg, Germany

[16]Department of Human Genetics, McGill University, Montreal, QC, Canada

[17]Division of Anatomical Pathology, British Columbia Children's Hospital and Women's Hospital and Health Center, Vancouver, British Columbia, Canada.

[18]University of British Columbia, Vancouver, British Columbia, Canada.

[19]Department of Pathology, Ghent University Hospital, Ghent, Belgium.

[20]Institute of Neuropathology, University Hospital Münster, Germany

[#]These authors contributed equally to this work.

Corresponding author:

Ulrich Schüller, MD

Research Institute Children's Cancer Center Hamburg

Martinistrasse 52, N63 (HPI), D-20251 Hamburg, Germany

Phone: +49-40-426051240

Fax: +49-40-741040350

E-mail: u.schueller@uke.de

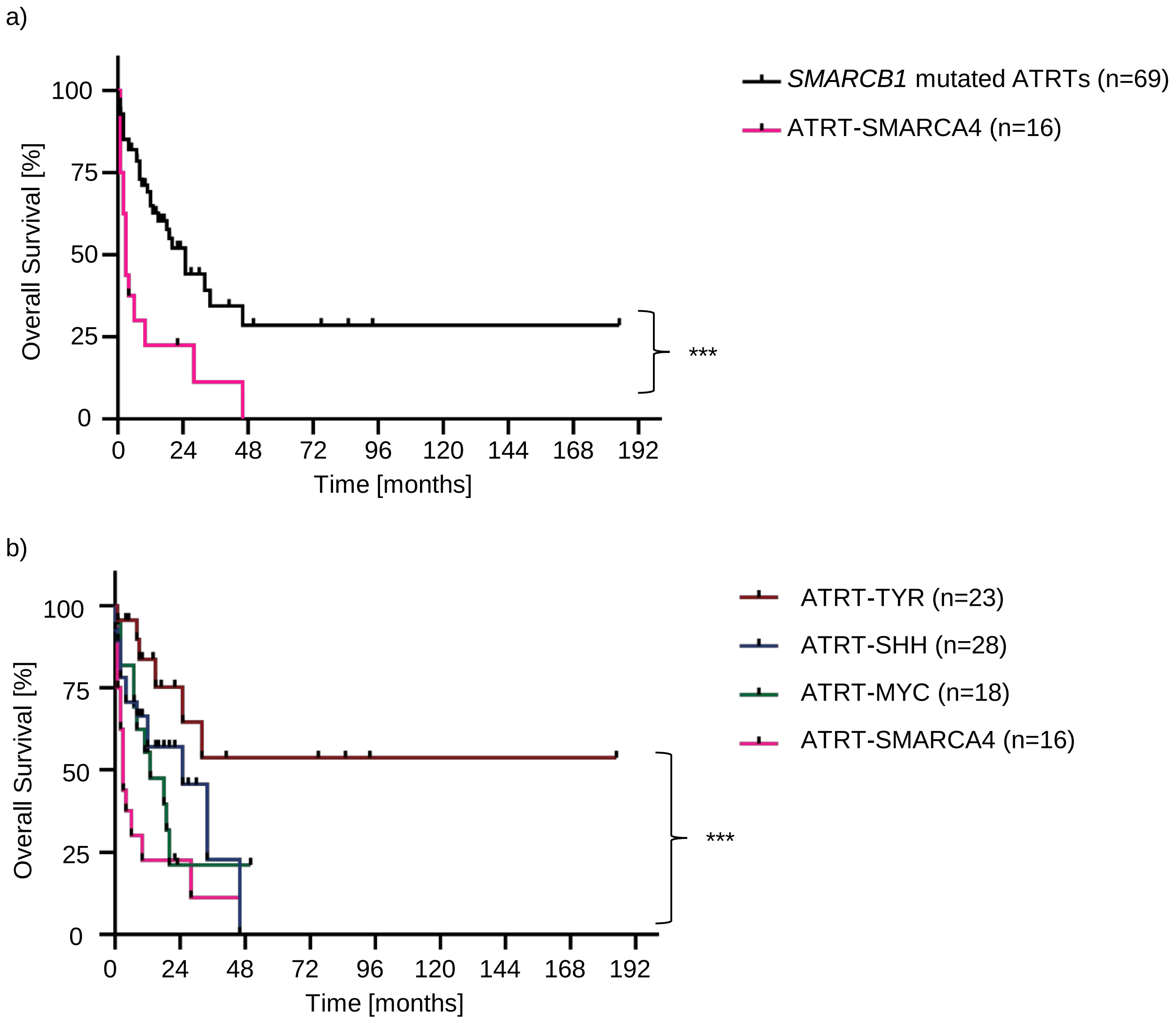

Supplementary Fig. 1
Kaplan Meier analysis for comparison of *SMARCB1* (n=69) and *SMARCA4* mutated ATRTs (n=16) shows significantly less survival in the latter (a). Kaplan Meier analysis for comparison of ATRT-TYR (n=23), ATRT-SHH (n=28), ATRT-MYC (n=18) and ATRT-SMARCA4 (n=16) depicts significant differences across all subgroups (b). Significance is based on a Log-Rank test. ***$p<0.001$. Note that this analysis included 5 published cases, for which tumor material was not available [5, 37].

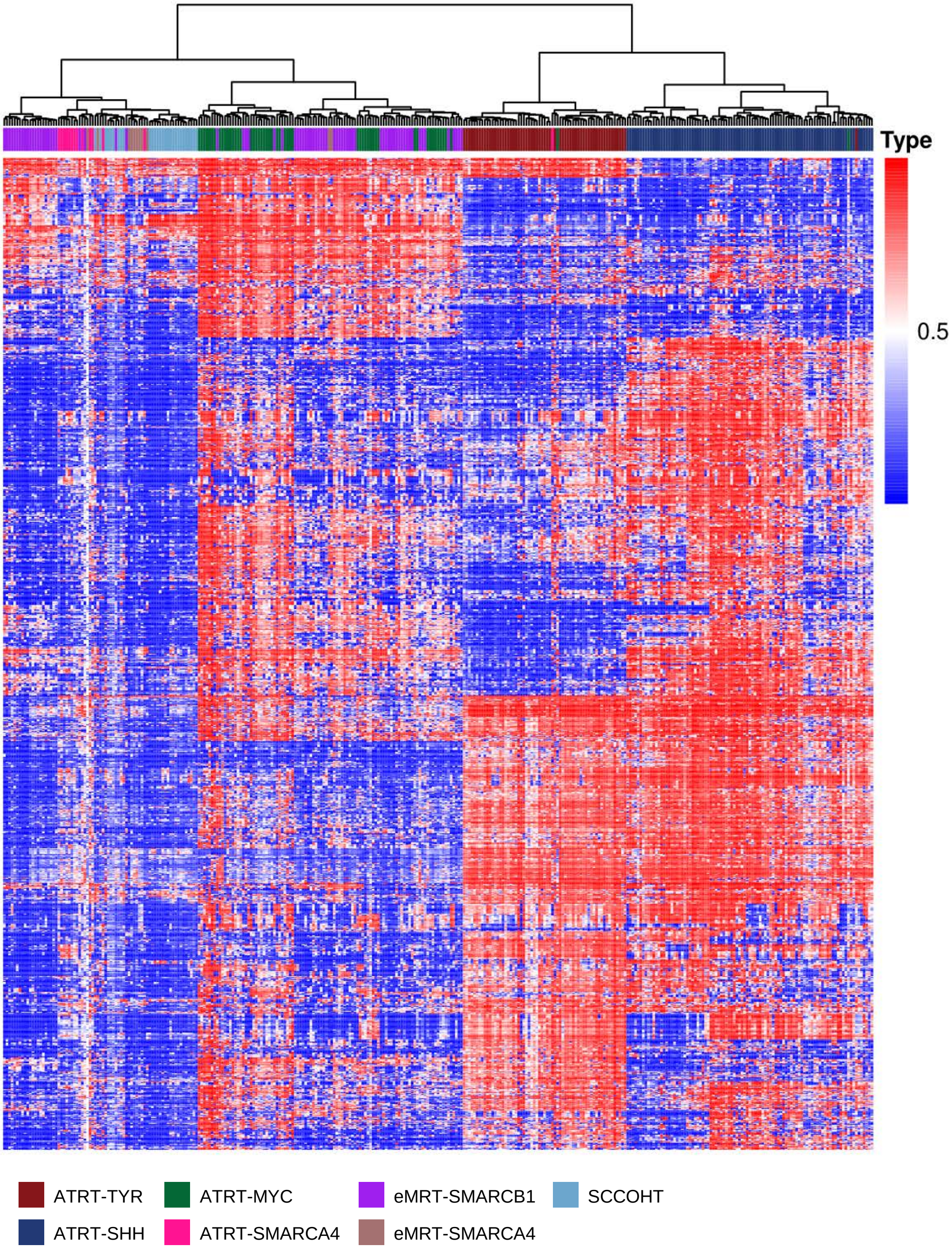

Supplementary Fig. 2
Unsupervised hierarchical clustering of n=14 ATRT-SMARCA4 (pink), n=11 eMRT-SMARCA4 (brown), n=62 ATRT-TYR (dark red), n=93 ATRT-SHH (blue), n=53 ATRT-MYC (dark green), n=74 eMRT-SMARCB1 (purple), and n=28 SCCOHTs (light blue) using the 1,000 most variant probes is shown. The level of DNA methylation is indicated by a color scale representing beta values.

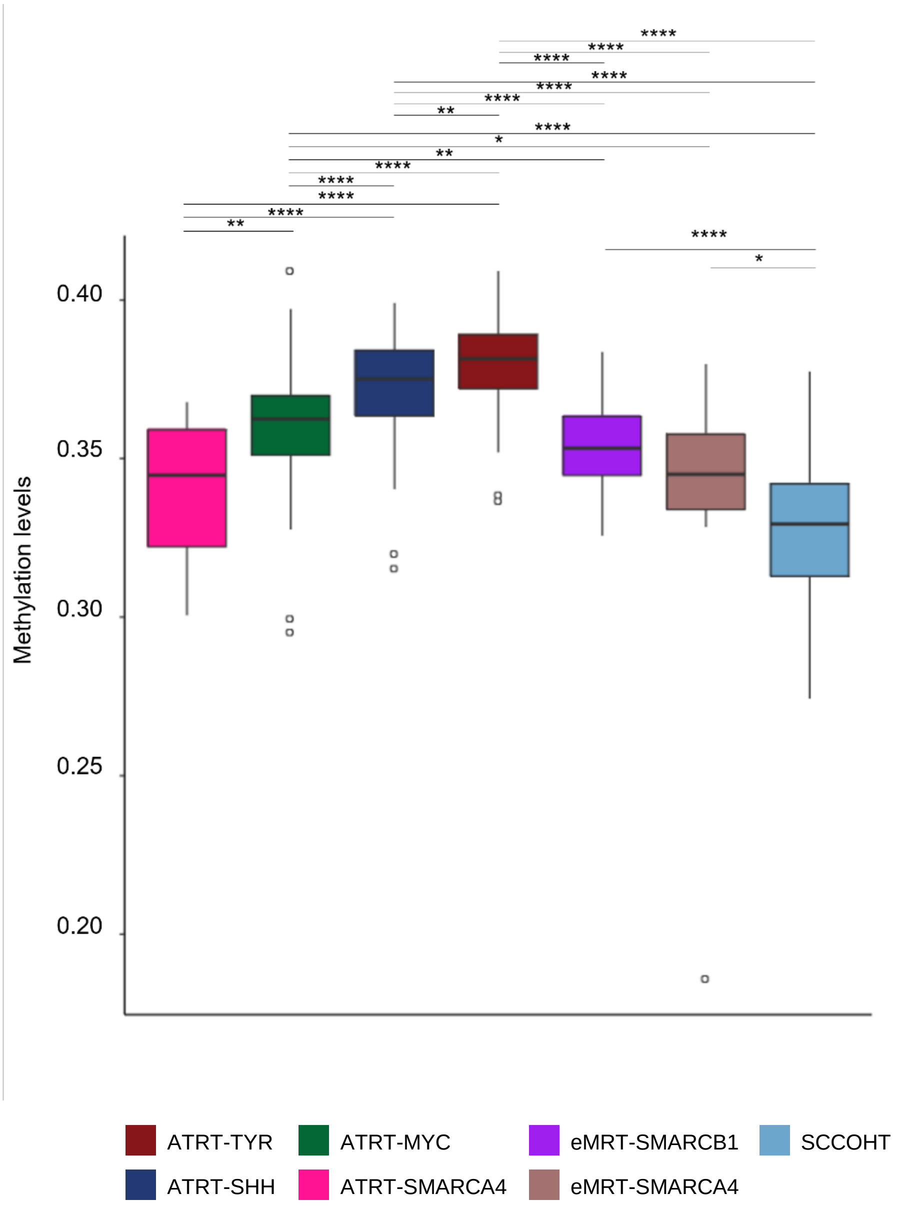

Supplementary Fig. 3

Genome-wide methylation levels of n=14 ATRT-SMARCA4 (pink), n=11 eMRT-SMARCA4 (brown), n=62 ATRT-TYR (dark red), n=93 ATRT-SHH (blue), n=53 ATRT-MYC (dark green), n=74 eMRT-SMARCB1 (purple), and n=28 SCCOHTs (light blue) are shown as boxplots where the y-axis indicates the β values derived from 450K/850K data. *p<0.05, **p>0.01, ***p>0.001, ***p>0.0001, not significant differences are not indicated.

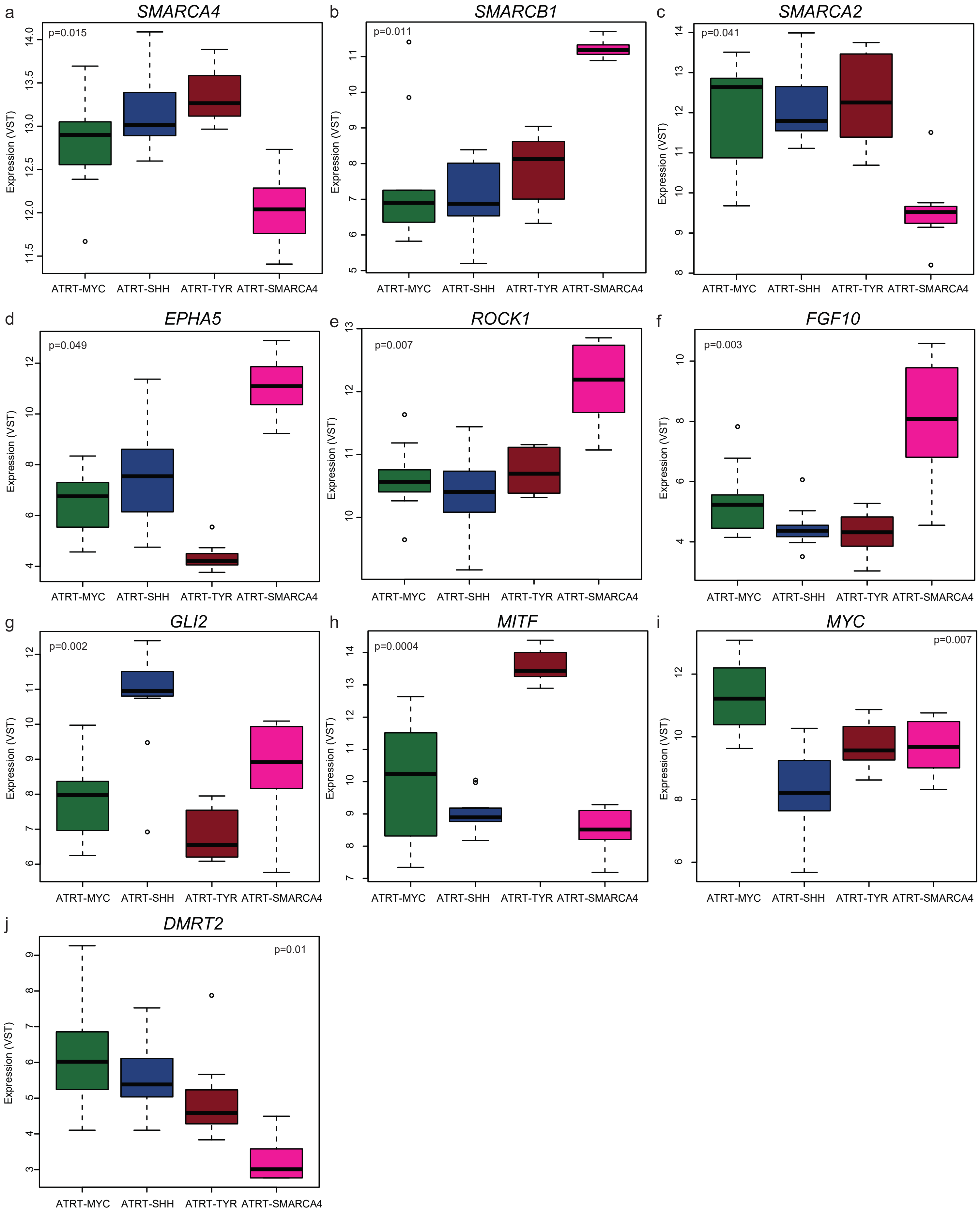

Supplementary Fig. 4
Relative expression levels of *SMARCA4* (a), *SMARCB1* (b), *SMARCA2* (c), *EPHA5* (d), *ROCK1* (e), *FGF10* (f), *GLI2* (g), *MITF* (h), *MYC* (i), and *DMRT2* (j) in the different ATRT subgroups are displayed as boxplots.

Supplementary Fig.5

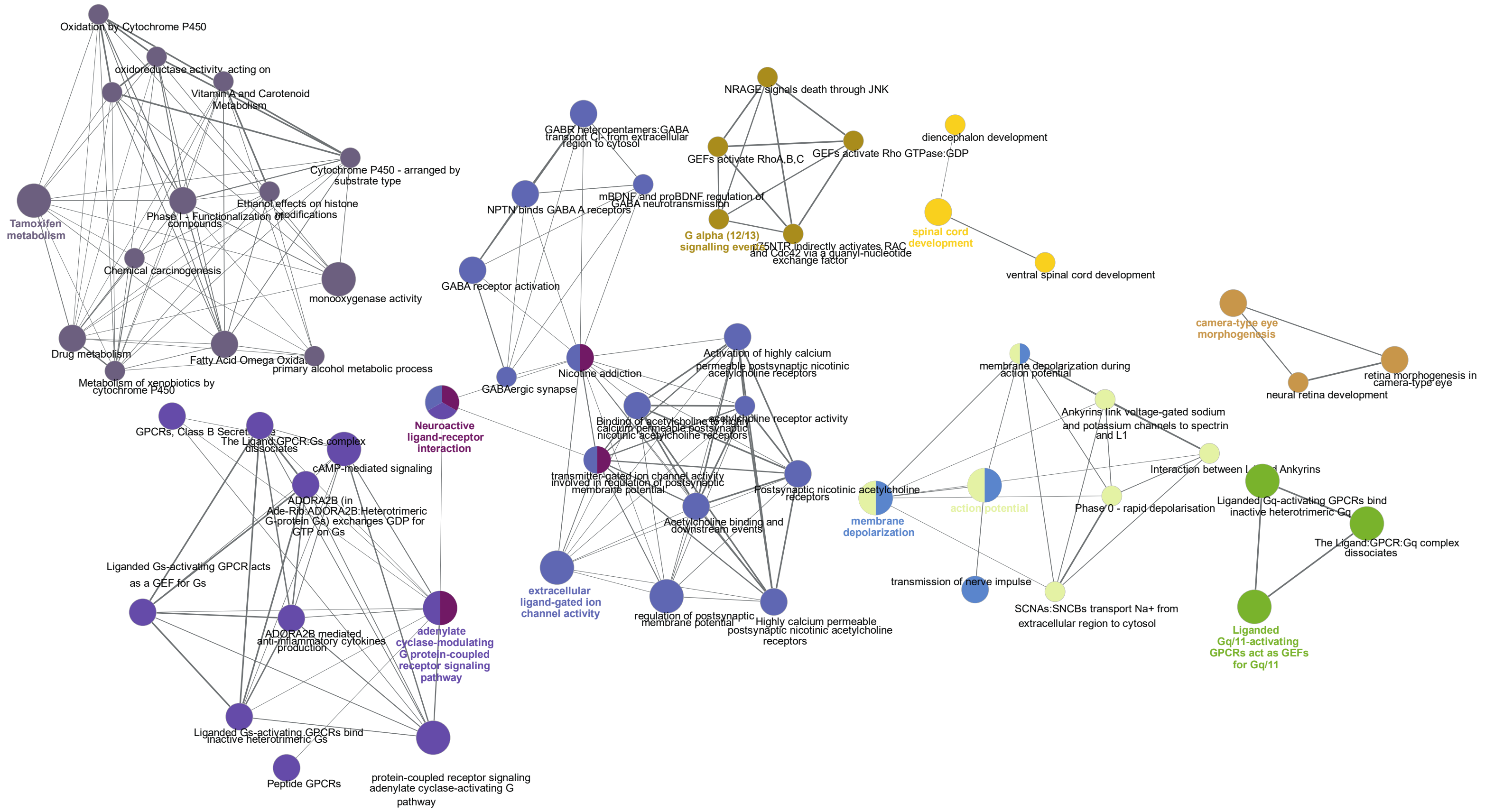

Supplementary Fig. 5

Network analysis of enriched terms in in *SMARCA4* mutated ATRTs compared to *SMARCB1* deficient ATRTs are depicted.

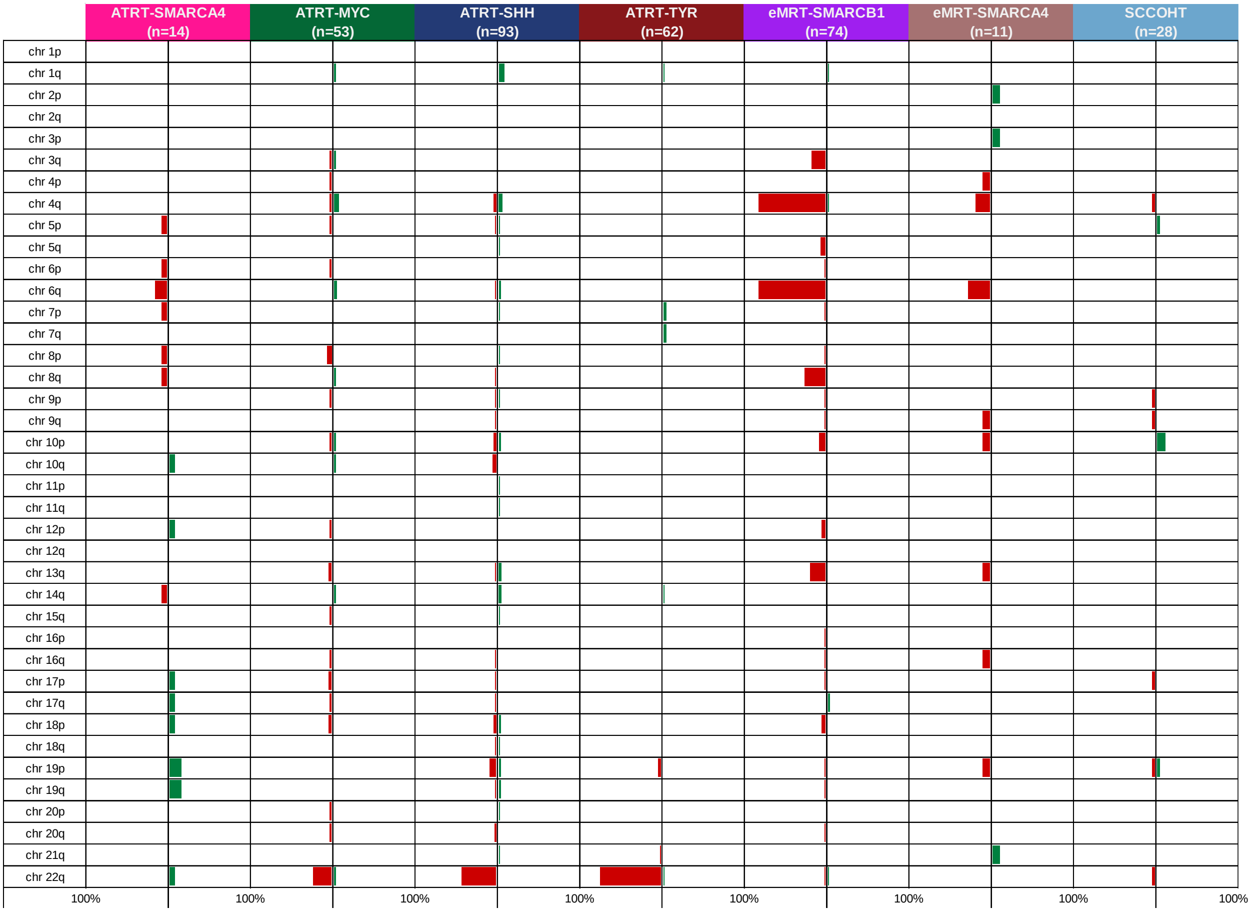

Supplementary Fig. 6

Overview of chromosomal gains and losses on q and p arms are shown as percentages of ATRT-SMARCA4 (n=14, pink), eMRT-SMARCA4 (n=11, brown), ATRT-TYR (n=62, dark red), ATRT-SHH (n=63, blue), ATRT-MYC (n=53, dark green), eMRT-SMARCB1 (n=74, purple), and SCCOHTs (n=28, light blue) calculated from 450K/850K DNA methylation data.

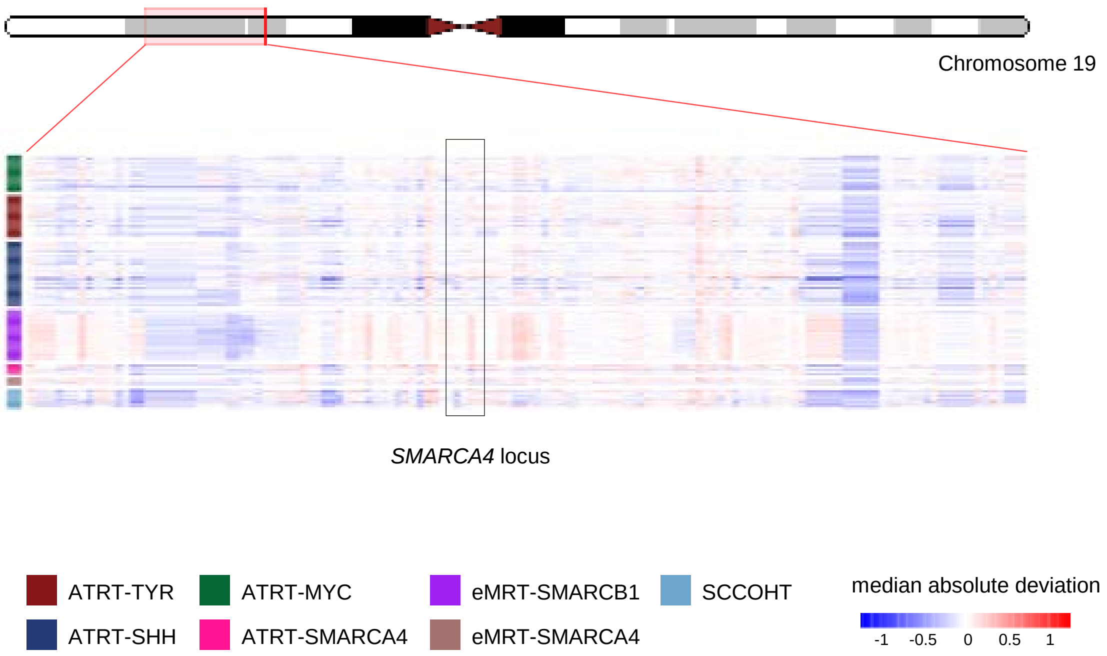

Supplementary Fig. 7
Chromosomal copy gains (red) and losses (blue) at the SMARCA4 locus inferred from 450K/850K DNA methylation data of ATRT-SMARCA4 (n=14, pink), eMRT-SMARCA4 (n=11, brown), ATRT-TYR (n=62, dark red), ATRT-SHH (n=63, blue), ATRT-MYC (n=53, dark green), eMRT-SMARCB1 (n=74, purple), and SCCOHTs (n=28, light blue) and summarized as a heatmap.

Supplementary Table 1: Overview of tumor samples used in this study.

**Type of Analysis**	***SMARCA4* mutated ATRTs**	***SMARCA4* mutated eMRTs**	***SMARCA4* mutated MBs**	***SMARCB1* mutated ATRT**	***SMARCB1* mutated eMRT**	***SMARCA4* wild type MB**	**SCCOHT**
Survival	n=11 present study<br>n=4 from [17]<br>n=1 from [2]			n=69 from [12]			
DNA methylation profiling	n=14 present study	n=10 present study<br>n=1 from [5]	n=2 present study<br>n=16 from [34]	n=12 present study<br>n=96 from [4,6,47]	n=74 from [6]	n=367 from [4]	n=1 present study<br>n=27 from [8]
RNA sequencing	n=8 present study			n=4 present study<br>n=24 from [22]			
Summary of all ATRT subgroups	n=14 present study<br>n=4 from [17]<br>n=1 from [2]			n≤321 from [19]<br>n=75 from [12]			

Supplementary Table 1: Overview of SMARCA4 mutated tumors without publicly available DNA methylation data prior to this study. infra: infratentorial, n/a, data not available, supra: supratentorial

Case No.	Tumor location	Diagnosis	Sex	Age at diagnosis [years]	Overall survival [years]	dead /alive	*SMARCA4* mutation in the tumor	Predicted AA change	Coding impact	Clinical significance (ClinVar)	Clinical significance (ACMG)	*SMARCA4* germline mutation	SMARC4 protein (IHC)	References
15	Brain (infra)	MB (Group 3)	male	8	n/a	n/a	n/a	p.H1097Q	missense	n/a	Uncertain significance	n/a	retained	
16	Brain (infra)	MB (WNT)	female	11	n/a	n/a	n/a	p.E821K	missense	n/a	Uncertain significance	n/a	retained	
17	Ovary	SCCOHT	female	14.4	n/a	n/a	c.728dup	p.A245Gfs*42	frameshift	n/a	Uncertain significance	n/a	n/a	
18	n/a	eMRT	n/a	n/a	n/a	dead	c.3168+1G>A	*Intron*	none	n/a	Pathogenic	yes	lost	
19	n/a	eMRT	male	n/a	n/a	n/a	c.4018G>A	p.E1340K	missense	n/a	Uncertain significance	n/a	lost	
20	Stomach	eMRT	female	4.5	n/a	alive	c.1943+1G>T	*Intron*	none	n/a	Pathogenic	yes	lost	
21	Kidney	eMRT	female	0.6	1.5	dead	c.3565C>T	p.R1189*	nonsense	Pathogenic	Pathogenic	yes	lost	Patient III3 [43]
22	Shoulder	eMRT	male	0.3	5	alive	n/a	n/a	n/a	n/a	n/a	n/a	lost	R907-18 [42]
23	Omentum	eMRT	male	0.4	<0.5	dead	n/a	n/a	n/a	n/a	n/a	n/a	lost	R907-05 [42]
24	n/a	eMRT	n/a	n/a	n/a	n/a	n/a	n/a	n/a	n/a	n/a	n/a	n/a	
25	n/a	eMRT	n/a	n/a	n/a	n/a	n/a	n/a	n/a	n/a	n/a	n/a	n/a	
26	n/a	eMRT	n/a	n/a	n/a	n/a	n/a	n/a	n/a	n/a	n/a	n/a	n/a	
27	n/a	eMRT	n/a	n/a	n/a	n/a	n/a	n/a	n/a	n/a	n/a	n/a	n/a	

Supplementary Table 1: Overview of unpublished SMARCB1 mutated ATRTs included in this study. infra: infratentorial, n/a, data not available, supra: supratentorial; √ analysis done; x analysis not done

Case No.	Tumor location	Sex	Age at diagnosis [months]	Molecular subgroup	DNA methylation data	RNA sequencing data
28	infra	male	8.4	ATRT-MYC	√	x
29	supra	male	118.8	ATRT-MYC	√	√
30	supra	male	18	ATRT-MYC	√	x
31	supra	male	2.4	ATRT-MYC	√	x
32	supra	male	30	ATRT-MYC	x	√
33	supra	male	38.4	ATRT-SHH	√	x
34	n/a	female	15.6	ATRT-SHH	√	x
35	n/a	male	13.2	ATRT-SHH	√	x
36	supra	male	60	ATRT-SHH	x	√
37	infra	male	12	ATRT-TYR	√	x
38	infra	female	31.2	ATRT-TYR	√	x
39	supra	male	19.2	ATRT-TYR	x	√
